# Assessing social accountability perspectives among Syrian medical students: a cross-sectional study

**DOI:** 10.1186/s12909-023-04969-9

**Published:** 2023-12-20

**Authors:** Sarya Swed, Hidar Alibrahim, Haidara Bohsas, Mohamad Nour Nasif, Yasmeen Abouainain, Yazan Khair Eldien Jabban, Eman Ali, Mohammad Badr Almoshantaf, Rana Alaa Alnajem, Rama Reslan, Tarek Majzoub, Bisher Sawaf, Wael Hafez

**Affiliations:** 1 Faculty of Medicine, Aleppo University, Aleppo, Syria; 2 Faculty of Medicine, University of Jordan, Amman, Jordan; 3 Faculty of Medicine, Damascus University, Damascus, Syria; 4 Dow University of Health Sciences, Karachi, Pakistan; 5 Faculty of Medicine, Tishreen University, Lattakia, Syria; 6 Department of Internal Medicine, Hamad Medical Corporation, Doha, Qatar; 7 NMC Royal Hospital, 16th Street, Khalifa City, Abu Dhabi, UAE

**Keywords:** Social, Accountability, Students, Syria, Cross-sectional

## Abstract

**Background:**

Social accountability (SA) within medical education signifies a commitment to address critical regional, societal, and national issues through educational, research, and service activities. In resource-limited regions, marginalized communities face barriers to accessing quality healthcare, and the concept of SA is often poorly understood by students. This study aims to investigate the perspectives, awareness, and comprehension of Syrian medical students regarding the concepts and principles of SA.

**Methods:**

This cross-sectional online study was conducted in Syria from June 1st to July 25th, 2023, to assess the perspectives on SA among medical students enrolled in pre-clinical and clinical phases from the 3rd to the 6th year, encompassing both stream I and stream II. The questionnaire included three parts: consent and introduction, socio-demographic data, and a 12-item survey assessing social accountability. Data were analyzed using Statistical Package for the Social Sciences software version 24 (SPSS 24).

**Results:**

A total of 1312 medical students (62.3% females vs. 37.7% males) participated in our analysis. Less than half of the participants (45.7%) reported that their institution had a limited social mission statement regarding the communities they serve. However, only 39.6% reported that their curriculum partially reflected the needs of the population they serve. A mere 7.5% and 6.8% of respondents indicated that their school had excellent community partners and stakeholders shaping their institution, and they learned significantly about other cultures and social circumstances in the medical context through their curriculum. About 24.1% reported that their institution required them to engage in a substantial amount of community-based learning, and 37.4% believed that their class reflected a good representation of socio-demographic characteristics of the reference population. A significant portion of the participants (44.3%) stated that their school did not encourage them to pursue generalist specialties, and 12.7% felt that their institution did not have a positive impact on the community. Among the included participants, 45.8% had some level of SA status, while 37.7% indicated good SA status. Age, gender, and the phase of study were the only sociodemographic characteristics statistically associated with SA status (p-value < 0.05). The association between the 12 items determining SA and the year of study was statistically significant for seven items (p-value < 0.05). However, adjusted logistic regression revealed no significant correlation between predicting SA status and sociodemographic factors (p-value > 0.05).

**Conclusion:**

This study underscores the significant influence of clinical experience and gender on Syrian medical students’ perceptions of SA. To enhance these perceptions, medical institutions should tailor support services for different stages of training and target initiatives to engage male students.

## Introduction

Social accountability (SA) in the context of medical education represents a profound commitment to addressing critical regional, societal, and national issues through a multifaceted approach encompassing educational, research, and service activities [1]. As the healthcare landscape continues to evolve, medical institutions are increasingly recognizing the imperative to adapt to the evolving priorities of patients, communities, and healthcare systems at various levels [2]. This necessitates a paradigm shift towards actively involving communities in shaping research and instructional initiatives. For instance, patient participation groups and curriculum review committees have emerged as crucial conduits for fostering a genuine sense of engagement and responsiveness within the medical education system.

To empower medical institutions to become socially accountable, a range of strategies is being explored. These strategies include the development of community-oriented admission policies and partnerships with underrepresented areas, which, in turn, serve to reinforce the institution’s connection with the community it serves. It’s essential to acknowledge that while there is mounting pressure on medical schools to demonstrate their dedication to social accountability on a global scale [3, 4], this task is riddled with complexities.

As healthcare professionals aim to enhance patients’ physical, emotional, and social well-being, the integration of social accountability within medical school curricula has gained increasing prominence [5]. In this era, where the medical profession is deeply ingrained in the ethos of healthcare service, SA is being viewed as a core component of medical school curricula more frequently.

Collaborative training initiatives, which emphasize population health and address health disparities, have played a pivotal role in equipping medical professionals with a profound understanding of social accountability and their responsibilities to their communities [6]. In resource-limited settings, such as Syria, marginalized communities often grapple with significant barriers to accessing quality healthcare, exacerbating health disparities. Despite the importance of SA, students’ understanding of this concept remains inadequate, contributing to a gap in the literature addressing the unique challenges faced by medical students in such contexts [7].

Existing literature emphasizes the growing recognition of social accountability’s importance in medical education [8]. Strategies, such as community-oriented admission policies and partnerships, have been explored, reflecting the complexities inherent in achieving social accountability [9]. Collaborative training initiatives addressing health disparities play a pivotal role in equipping medical professionals with the necessary skills and perspectives [10].

However, the literature reveals persistent gaps in understanding the specific challenges faced by medical students in resource-limited regions like Syria. While prior research touches on the importance of SA, there is a dearth of studies exploring these issues in non-Western contexts, contributing to our rationale for this study.

This study is situated within the Social Cognitive Theory (SCT) proposed by Bandura [11]. SCT emphasizes the dynamic interplay between personal factors, environmental influences, and behavior. Applied to medical education and social accountability, SCT allows us to understand how the perceptions of medical students are shaped by individual experiences and environmental factors. It provides a theoretical context for comprehending how exposure to community-oriented policies, collaborative training initiatives, and institutional connections with underrepresented areas influence the development of social accountability attitudes among students.

This study delves into the perspectives of Syrian medical students on the principles of social accountability, aiming to unravel the complexities of their awareness and comprehension. In contrast to existing studies, our research focuses on a region with distinct healthcare challenges, where SA is often poorly understood. By investigating the perceptions of medical students in Syria, we aim to contribute nuanced insights into the intersection of medical education, societal needs, and the role of healthcare professionals in resource-limited settings.

While prior research has touched upon the importance of SA, our study stands out by addressing the specific context of Syrian medical education, shedding light on the unique challenges and opportunities in a region undergoing profound social changes. The existing literature predominantly focuses on Western contexts, and the dearth of research in regions with different socio-economic and healthcare environments underscores the significance of our exploration.

## Methods

### Study design and setting

This cross-sectional online study was conducted in Syria from June 1st to July 25th, 2023, to comprehensively assess the perspectives, awareness, and comprehension of Syrian medical students regarding the concepts and principles of social accountability. The chosen timeframe aligns strategically with the academic calendar of Syrian medical institutions, ensuring comprehensive representation across various academic years. Conducting the study during this period allows us to capture a diverse range of experiences, including responses from students engaged in both pre-clinical and clinical phases, providing a holistic view of their perceptions of social accountability.

#### Inclusion and exclusion criteria

The study included medical students currently enrolled in both clinical and preclinical phases, spanning from the third to the sixth year. Eligibility extended to participants from both Stream I (recent high school graduates) and Stream II (bachelor’s degree holders in science, applied medical science, and pharmacy enrolled in medicine as a second major). Medical students in the 3rd to 6th year were included in the study due to their advanced stage in medical education, where exposure to clinical settings and community engagement is more pronounced. The decision to focus on these years is rooted in the assumption that students in later stages of their medical education would have gained sufficient exposure to contextualize and reflect on social accountability principles within the framework of their training.

#### Participant information and consent

All participants received detailed explanations about the study’s objectives, the research team involved, and their rights concerning confidentiality and the option to withdraw from the study. Only fully completed submissions were accepted and assessed to ensure data quality and reliability.

#### Questionnaire development

The questionnaire used in this study was developed in collaboration between the International Federation of Medical Students Association (IFMSA) and the Training for Health Equity Network (THEnet) [12]. To ensure the questionnaire’s comprehensibility to respondents, it underwent a meticulous translation process into Arabic. Certified and professional translators oversaw the translation, and any disagreements were resolved through consensus.

#### Data collection strategies

Data collection employed a combination of convenience and snowball tactics. The questionnaire was hosted on Google Forms and distributed to participants through various social media platforms, including Facebook, Twitter, WhatsApp, and others.

#### Sample size

The minimal sample size was found by applying a single proportion of the population formula [n = [(Zα/2)². P (1-P)]/d²]. With a 95% confidence level (Zα/2 = 1.96), a 5% margin of error, and a population proportion of 50% to ensure the largest sample size, the final size of the sample required was 385. A total of 1318 participants contributed to the sample, providing a diverse representation of medical students in Syria.

### Measures

The questionnaire employed in this study comprised three parts. Part 1 provided a brief introduction to the study’s aim and sought participants’ consent to participate. Part 2 encompassed 11 questions related to the socio-demographic characteristics of the study population. These questions covered aspects such as gender, age, current year of study, clinical phase, stream, cumulative GPA in the past year, nationality of the teaching faculty, familial affiliations with healthcare professions, and the name of the participant’s university.

Part 3 involved 12 questions employing the IFMSA and THEnet 12-item survey to assess medical faculties’ social accountability from the perspective of medical students. This survey evaluated various aspects of social accountability in medical education, including the presence of populations served by medical college students during their practical training, the positive impact of the college on the community, community-based research conducted by the college, encouragement of students to pursue generalist specialties, inclusion of community-based learning in the school’s learning objectives, consideration of other cultures in a medical context in the educational program, responsiveness to societal demands, and the extent to which the college allows students to play an active role in assisting their community. Each question was assessed using a four-point Likert scale (no = 0, somewhat = 1, good = 2, excellent = 3), resulting in a range of scores from 0 to 36 for the 12 questions. The total score was subsequently categorized into four groups: limited infrastructure in Social Accountability (score: 0–8), some Social Accountability (score: 9–17), areas for improvement (score: 18–26), and substantial infrastructure in Social Accountability (score: 27–36).

### Pilot study

To validate the Arabic version of the questionnaire and ensure its reliability and coherence, we conducted a pilot study. The survey was pre-tested by distributing it among 50 randomly selected participants from the pool of medical students included in the original study. It is crucial to note that these participants were subsequently excluded from the final sample to prevent duplication.

The primary objective of the pilot study was to assess the internal consistency and reliability of the Arabic version of the survey. Statistical measures, including Cronbach’s alpha, were employed for this purpose. The obtained value of Cronbach’s alpha was above 0.712, indicating a high degree of internal consistency and affirming the questionnaire’s reliability and coherence in the Arabic language.

Following the successful completion of the pilot test and the confirmation of the questionnaire’s reliability, we proceeded with the widespread distribution of the survey among the intended study participants.

### Ethical consideration

Ethical approval for this study was granted by the Syrian Ethical Society for Scientific Research in Aleppo (IRB: 33/MO). Informed consent was obtained through a standard question at the start of the survey, and participants who agreed to participate were directed to complete the detailed study questions. The survey took approximately 10–15 min to complete, and all responses were securely stored in an online database.

### Statistical analysis

Data entry and encoding were initially conducted using a Microsoft Excel spreadsheet, with rigorous checks performed to ensure data accuracy and address instances of missing information. The dataset was subsequently transferred for statistical analysis using Statistical Package for the Social Sciences software version 24 (SPSS 24). Descriptive statistics, including means and standard deviations (SDs), were computed and reported for all variables. Categorical data were presented as proportions or percentages.

The cumulative score derived from the 12 social accountability (SA)-related items was calculated and presented as mean ± SD for individual items. To provide a meaningful interpretation of the overall SA foundation, this total score was categorized into four distinct groups: limited SA foundation (score: 0–8), moderate SA foundation (score: 9–17), identification of areas for enhancement (score: 18–26), and robust SA foundation (score: 27–36). The proportional representation of each SA category was also reported.

In the exploration of the relationship between SA categories and student profiles, relevant statistical tests, such as chi-square and Fisher’s exact tests where applicable, were employed. To further enhance the analysis, a novel variable was introduced, aggregating the scores from the SA items. A threshold of ≥ 18 was set to indicate an acceptable level of social accountability, while scores ≤ 17 were classified as reflecting a lower level.

Binary logistic regression was employed to further analyze the relationship between social accountability categories and student profiles. This method allows for the examination of the influence of various independent variables on the likelihood of achieving a certain outcome, in this case, different levels of social accountability. All relevant variables were included in the binary logistic regression equation to ensure a comprehensive analysis. This approach allows for a nuanced understanding of the impact of different factors on social accountability outcomes among the study participants.

### Rationale for threshold selection

The selection of this specific threshold is grounded in the comprehensive nature of the SA scale, which spans a range of potential scores from 0 to 36. A threshold of ≥ 18 was considered indicative of acceptable SA to ensure a balanced representation of the participants’ commitment to social accountability principles. This demarcation was established through careful consideration of the distribution of scores and aligns with existing literature on SA assessment in medical education [13]. The adoption of this threshold allows for a clearer interpretation of the results and facilitates meaningful comparisons across different levels of SA commitment among the study participants.

Statistical significance for all conducted tests was defined as p-values < 0.05, ensuring a robust analysis of the relationships and patterns observed within the dataset.

## Results

In this section, we present the findings of our study, which aimed to explore the perspectives, awareness, and comprehension of Syrian medical students concerning the concepts and principles of social accountability. The results offer insights into the levels of social accountability among participants, shedding light on the dynamics within medical education in the context of a resource-limited setting.

### Demographic profile of study participants

A total of 1318 medical students were initially considered for this study; however, six declined to participate, resulting in a final sample size of 1312 participants (99.5%). The gender distribution among participants showed that 62.3% were females, while 42.9% fell within the age range of 22 to 25 years. Regarding the academic year, 36.1% of respondents reported being in their fourth year, and 68.4% were in the clinical phase of their studies. Only 8.7% of respondents had a GPA above 90%, and 20.4% indicated having parents who belonged to the medical sector. Lastly, the majority of participants (95.5%) were affiliated with Government universities. Detailed characteristics of the included participants can be found in Table 1.

**Table 1:** Demographic profile of study participants

		Count	Layer N %
Gender	Female	817	62.3%
	Male	495	37.7%
Age	18–21	721	55.4%
	22–25	558	42.9%
	26–29	20	1.5%
	30<	2	0.2%
Year of study	3rd year	362	27.6%
	Fourth year	474	36.1%
	Fifth year	252	19.2%
	Final year	224	17.1%
Phase of study	Pre-clinical	415	31.6%
	Clinical	897	68.4%
Entry level	School entrant	1282	97.7%
	Graduate entrant	30	2.3%
GPA	60–70	71	5.4%
	71–80	457	34.8%
	81–90	670	51.1%
	90<	114	8.7%
Teaching faculty	Syrians	1305	99.5%
	Non-Syrians	7	0.5%
Parents background	No	1045	79.6%
	Yes	267	20.4%
Sibling background	No	861	65.6%
	Yes	451	34.4%
Type of university	Governmental	1237	95.5%
	Private	58	4.5%

### Perceptions of social accountability among medical students

As seen in Table 2, less than half of the participants (45.7%) reported that their institution had a limited social mission statement regarding the communities they serve. Additionally, 39.6% of respondents stated that their curriculum only partially reflected the needs of the population they serve. A small proportion of the study sample (7.5%) believed their school had excellent community partners and stakeholders who played a role in shaping their institution, while an equally small percentage (6.8%) felt that they learned excellently about other cultures and different social circumstances in the medical context within their curriculum.

Among the 1312 candidates included in our study, only 24.1% reported that their institution required them to engage in a significant amount of community-based learning. On the other hand, 37.4% of students reported that their class reasonably represented the socio-demographic characteristics of the reference population.

Conversely, a notable portion (44.3%) of the participants stated that their school did not actively encourage them to pursue generalist specialties such as family medicine or general practice. Furthermore, 12.7% of respondents expressed that their school did not have a positive impact on the community.

**Fig. 1:**
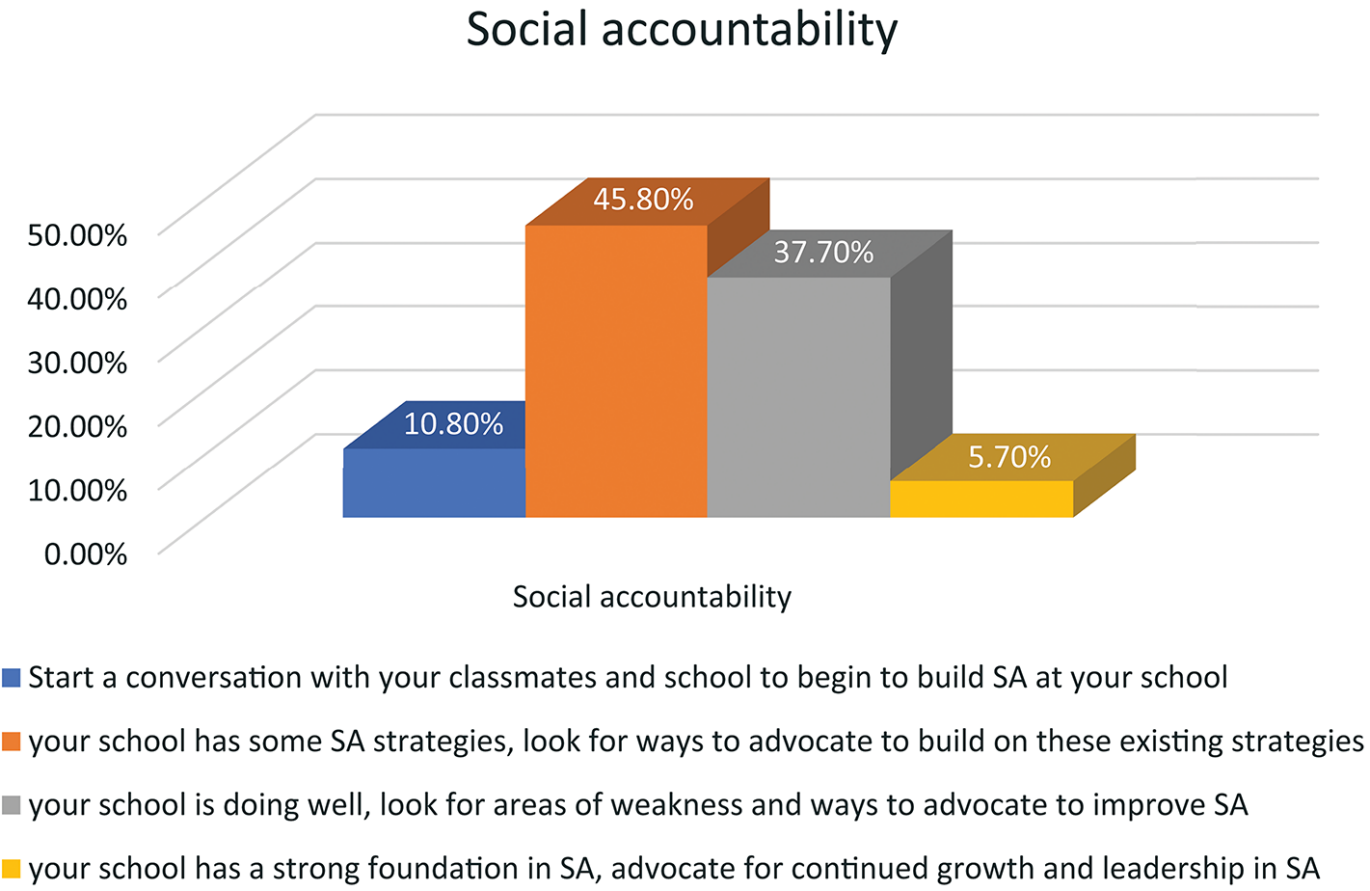
Social accountability status

Figure 1 reveals that 45.8% of participants demonstrated some degree of social accountability status, whereas 37.7% indicated a relatively higher level of social accountability.

**Table 2:** Descriptive summary of the items determining social accountability

		Frequency	Percentage %	Mean	Std. deviation
Does your institution have a clear social mission (statement) around the communities that they serve?	No	271	20.7%	1.20	0.84
	A little bit	600	45.7%		
	Good	354	27.0%		
	Excellent	87	6.6%		
Does your curriculum reflect the needs of the population you serve?	No	165	12.6%	1.47	0.85
	A little bit	519	39.6%		
	Good	478	36.4%		
	Excellent	150	11.4%		
Does your school have community partners and stakeholders who shape your school?	No	409	31.2%	1.1	0.92
	A little bit	499	38.0%		
	Good	305	23.2%		
	Excellent	99	7.5%		
Do you learn about other cultures and other social circumstances in medical context in your curriculum?	No	329	25.1%	1.17	0.88
	A little bit	522	39.8%		
	Good	372	28.4%		
	Excellent	89	6.8%		
Do the places/locations you learn at in practice include the presence of the populations that you will serve?	No	205	15.6%	1.63	0.98
	A little bit	350	26.7%		
	Good	479	36.5%		
	Excellent	278	21.2%		
Are you required to do community-based learning (opposed to only elective opportunities)?	No	419	31.9%	1.1	0.95
	A little bit	463	35.3%		
	Good	316	24.1%		
	Excellent	114	8.7%		
Does your class reflect the socio—demographic characteristics of your reference population?	No	76	5.8%	2.02	0.89
	A little bit	282	21.5%		
	Good	491	37.4%		
	Excellent	463	35.3%		
Do your teachers reflect the socio—demographic characteristics of your reference population?	No	142	10.8%	1.57	0.89
	A little bit	497	37.9%		
	Good	452	34.5%		
	Excellent	221	16.8%		
Does your learning experience also provide an active service to your community?	No	247	18.8%	1.4	0.94
	A little bit	464	35.4%		
	Good	429	32.7%		
	Excellent	172	13.1%		
Does your school have community-based research?	No	318	24.2%	1.23	0.91
	A little bit	480	36.6%		
	Good	406	30.9%		
	Excellent	108	8.2%		
Does your school encourage you to undertake generalist specialities (e.g., family medicine, general practice)?	No	581	44.3%	0.91	0.98
	A little bit	385	29.3%		
	Good	233	17.8%		
	Excellent	113	8.6%		
Does your school have a positive impact on the community?	No	167	12.7%	1.57	0.92
	A little bit	450	34.3%		
	Good	471	35.9%		
	Excellent	224	17.1%		

### Association of profile characteristics with SA status in respective institutions

In Table 3, out of the ten sociodemographic characteristic variables analyzed, only three showed a statistically significant association with SA status (p-value < 0.05). Notably, 25.1% of female participants exhibited a good SA status, while only 2.4% of male participants achieved an excellent SA status. Within the age group of 22–25 years, 20.2% of participants demonstrated some SA status. However, among respondents aged 18–21 years, 21.4% indicated a good SA status.

This table summarizes the association between participants’ sociodemographic characteristics and their SA status within their institutions, highlighting significant differences based on gender and age groups.

**Table 3:** Association of the profile of participants with SA of the institution

		Social accountability status								P-value
		No		Sometime		Good		Excellent		
		Count	Layer N %	Count	Layer N %	Count	Layer N %	Count	Layer N %	
Gender	Female	79	6.0%	366	27.9%	329	25.1%	43	3.3%	0.049
	Male	63	4.8%	235	17.9%	165	12.6%	32	2.4%	
Age	18–21	70	5.4%	325	25.0%	278	21.4%	48	3.7%	0.047
	22–25	70	5.4%	263	20.2%	201	15.4%	24	1.8%	
	26–29	0	0.0%	9	0.7%	10	0.8%	1	0.1%	
	30<	0	0.0%	1	0.1%	0	0.0%	1	0.1%	
Year of study	3rd year	44	3.4%	170	13.0%	120	9.1%	28	2.1%	0.077
	Fourth year	44	3.4%	205	15.6%	201	15.3%	24	1.8%	
	Fifth year	31	2.4%	109	8.3%	100	7.6%	12	0.9%	
	Final year	23	1.8%	117	8.9%	73	5.6%	11	0.8%	
Phase of study	Pre-clinical	53	4.0%	191	14.6%	140	10.7%	31	2.4%	0.049
	Clinical	89	6.8%	410	31.3%	354	27.0%	44	3.4%	
Entry level	School entrant	138	10.5%	587	44.7%	484	36.9%	73	5.6%	0.94
	Graduate entrant	4	0.3%	14	1.1%	10	0.8%	2	0.2%	
GPA	60–70	12	0.9%	32	2.4%	23	1.8%	4	0.3%	0.88
	71–80	51	3.9%	204	15.5%	173	13.2%	29	2.2%	
	81–90	68	5.2%	312	23.8%	253	19.3%	37	2.8%	
	90<	11	0.8%	53	4.0%	45	3.4%	5	0.4%	
Teaching faculty	Syrians	140	10.7%	598	45.6%	493	37.6%	74	5.6%	0.26
	Non-Syrians	2	0.2%	3	0.2%	1	0.1%	1	0.1%	
Parents background	No	114	8.7%	473	36.1%	392	29.9%	66	5.0%	0.31
	Yes	28	2.1%	128	9.8%	102	7.8%	9	0.7%	
Sibling background	No	100	7.6%	387	29.5%	321	24.5%	53	4.0%	0.43
	Yes	42	3.2%	214	16.3%	173	13.2%	22	1.7%	
Type of university	Governmental	133	10.3%	573	44.2%	463	35.8%	68	5.3%	0.37
	Private	8	0.6%	20	1.5%	26	2.0%	4	0.3%	

### Association between 12 items determining SA and year of study

The association between the 12 items determining Social Accountability (SA) and the year of study has been analyzed, with statistical significance observed for seven items (p-value < 0.05). Notably, 10.3% of students in the fifth year and 4.9% in the fourth year reported that their institution has a clear and good social mission statement regarding the communities they serve. Among final-year students, 7.3% indicated that their curriculum partially reflects the needs of the population they serve, while 3.7% of third-year students expressed that their curriculum reflects this aspect excellently. Additionally, 3.1% of fourth-year participants highlighted that the places/locations where they learn in practice did not include the presence of the populations they will serve. Furthermore, 11.2%, 13.3%, and 7.9% of participants in the third, fourth, and fifth year, respectively, emphasized that their teachers only partially reflect the socio-demographic characteristics of the reference population.

For further details and a comprehensive overview, please refer to Table 4.

**Table 4:** Association of the 12 items determining SA with the year of study

		Year of study								P-value
		3rd year		Fourth year		Fifth year		Final year		
		Count	Layer N %	Count	Layer N %	Count	Layer N %	Count	Layer N %	
Does your institution have a clear social mission (statement) around the communities that they serve?	No	70	5.3%	94	7.2%	60	4.6%	47	3.6%	0.04
	A little bit	156	11.9%	225	17.1%	112	8.5%	107	8.2%	
	Good	97	7.4%	135	10.3%	64	4.9%	58	4.4%	
	Excellent	39	3.0%	20	1.5%	16	1.2%	12	0.9%	
Does your curriculum reflect the needs of the population you serve?	No	37	2.8%	52	4.0%	38	2.9%	38	2.9%	0.032
	A little bit	133	10.1%	183	13.9%	107	8.2%	96	7.3%	
	Good	143	10.9%	189	14.4%	78	5.9%	68	5.2%	
	Excellent	49	3.7%	50	3.8%	29	2.2%	22	1.7%	
Does your school have community partners and stakeholders who shape your school?	No	119	9.1%	143	10.9%	77	5.9%	70	5.3%	0.91
	A little bit	134	10.2%	172	13.1%	103	7.9%	90	6.9%	
	Good	83	6.3%	120	9.1%	53	4.0%	49	3.7%	
	Excellent	26	2.0%	39	3.0%	19	1.4%	15	1.1%	
Do you learn about other cultures and other social circumstances in medical context in your curriculum?	No	97	7.4%	108	8.2%	61	4.6%	63	4.8%	0.22
	A little bit	128	9.8%	188	14.3%	112	8.5%	94	7.2%	
	Good	107	8.2%	142	10.8%	66	5.0%	57	4.3%	
	Excellent	30	2.3%	36	2.7%	13	1.0%	10	0.8%	
Do the places/locations you learn at in practice include the presence of the populations that you will serve?	No	125	9.5%	41	3.1%	25	1.9%	14	1.1%	0.000
	A little bit	108	8.2%	111	8.5%	69	5.3%	62	4.7%	
	Good	93	7.1%	191	14.6%	102	7.8%	93	7.1%	
	Excellent	36	2.7%	131	10.0%	56	4.3%	55	4.2%	
Are you required to do community-based learning (opposed to only elective opportunities?	No	133	10.1%	153	11.7%	76	5.8%	57	4.3%	0.002
	A little bit	101	7.7%	158	12.0%	103	7.9%	101	7.7%	
	Good	100	7.6%	121	9.2%	50	3.8%	45	3.4%	
	Excellent	28	2.1%	42	3.2%	23	1.8%	21	1.6%	
Does your class reflect the socio—demographic characteristics of your reference population?	No	23	1.8%	20	1.5%	9	0.7%	24	1.8%	0.03
	A little bit	83	6.3%	103	7.9%	53	4.0%	43	3.3%	
	Good	136	10.4%	169	12.9%	106	8.1%	80	6.1%	
	Excellent	120	9.1%	182	13.9%	84	6.4%	77	5.9%	
Do your teachers reflect the socio—demographic characteristics of your reference population?	No	36	2.7%	53	4.0%	17	1.3%	36	2.7%	0.08
	A little bit	147	11.2%	174	13.3%	103	7.9%	73	5.6%	
	Good	124	9.5%	158	12.0%	93	7.1%	77	5.9%	
	Excellent	55	4.2%	89	6.8%	39	3.0%	38	2.9%	
Does your learning experience also provide an active service to your community?	No	67	5.1%	96	7.3%	47	3.6%	37	2.8%	0.28
	A little bit	114	8.7%	168	12.8%	93	7.1%	89	6.8%	
	Good	121	9.2%	159	12.1%	76	5.8%	73	5.6%	
	Excellent	60	4.6%	51	3.9%	36	2.7%	25	1.9%	
Does your school have community-based research?	No	88	6.7%	98	7.5%	68	5.2%	64	4.9%	0.06
	A little bit	119	9.1%	185	14.1%	89	6.8%	87	6.6%	
	Good	114	8.7%	152	11.6%	79	6.0%	61	4.6%	
	Excellent	41	3.1%	39	3.0%	16	1.2%	12	0.9%	
Does your school encourage you to undertake generalist specialties (e.g., family medicine, general practice)?	No	161	12.3%	217	16.5%	100	7.6%	103	7.9%	0.75
	A little bit	109	8.3%	130	9.9%	84	6.4%	62	4.7%	
	Good	61	4.6%	85	6.5%	50	3.8%	37	2.8%	
	Excellent	31	2.4%	42	3.2%	18	1.4%	22	1.7%	
Does your school have a positive impact on the community?	No	49	3.7%	52	4.0%	33	2.5%	33	2.5%	0.001
	A little bit	101	7.7%	167	12.7%	89	6.8%	93	7.1%	
	Good	125	9.5%	185	14.1%	91	6.9%	70	5.3%	
	Excellent	87	6.6%	70	5.3%	39	3.0%	28	2.1%	

### Logistic regression analysis of factors influencing social accountability status among medical students

In our adjusted logistic regression analysis, we did not observe any significant correlation between predicting Social Accountability (SA) status and sociodemographic factors (p-value > 0.05). However, in a non-adjusted logistic regression analysis of our results, we found that gender was a significant predictor of SA status (p-value < 0.05). Specifically, males were less likely to exhibit adequate social accountability status compared to females (OR = 0.791). Further details regarding binary logistic regression between the baseline characteristics of the study population and SA status can be found in Table 5.

**Table 5:** Binary logistic regression between baseline characteristics of the study population and social accountability status

		P-value	Adjusted odds ratio	Lower	Upper	P-value	Non-adjusted odds ratio	Lower	Upper
Gender	Female								
	Male	0.062	0.800	0.633	1.012	0.042	0.791	0.630	0.992
Age	18–21	0.478				0.239			
	22–25	0.365	0.852	0.603	1.205	0.080	0.819	0.654	1.024
	26–29	0.336	1.605	0.612	4.209	0.389	1.481	0.606	3.617
	30<	0.943	1.107	0.067	18.401	0.892	1.212	0.075	19.447
Year of study	3rd Year	0.303				0.060			
	Fourth year	0.309	1.343	0.761	2.370	0.058	1.307	0.991	1.723
	Fifth year	0.409	1.315	0.687	2.518	0.380	1.157	0.836	1.601
	Final year	0.959	0.983	0.499	1.933	0.416	0.868	0.616	1.222
Phase of study	Pre-clinical								
	Clinical	0.987	1.004	0.592	1.703	0.282	1.138	0.899	1.441
Entry level	School entrant								
	Graduate entrant	0.846	0.924	0.417	2.049	0.707	0.868	0.415	1.816
GPA	60–70	0.699				0.810			
	71–80	0.252	1.378	0.797	2.384	0.330	1.291	0.772	2.157
	81–90	0.315	1.323	0.766	2.286	0.395	1.244	0.752	2.056
	90<	0.289	1.422	0.742	2.728	0.434	1.273	0.695	2.332
Teaching faculty	Syrians								
	Non-Syrians	0.476	0.546	0.103	2.882	0.436	0.521	0.101	2.693
Parents background	No								
	Yes	0.789	0.960	0.715	1.290	0.507	0.912	0.695	1.197
Sibling background	No								
	Yes	0.697	0.951	0.740	1.223	0.944	0.992	0.788	1.248
Type of university	Governmental								
	Private	0.063	1.711	0.971	3.015	0.188	1.425	0.841	2.413
Constant		0.052	0.566						

The logistic regression model wasn’t statistically significant, X2(16) = 18.003198, p-value = 0.323710

Hosmer and lemeshow test: 6.454712 (P-value = 0.596435)

The model explained 1.9% (Nagelkerke R Square) of the variance in social accountability status

## Discussion

Social accountability, often referred to as the social contract, represents the reciprocal obligations and responsibilities shared between the medical field and society at large. It is essential that social accountability is made accessible to all individuals equitably and is responsive to the healthcare needs of patients, communities, and the broader population. Prioritizing specific health issues is a collective effort, shaped by the perspectives of the public, government bodies, healthcare organizations, and healthcare professionals.

This study aimed to explore the viewpoints of Syrian medical students regarding the practice of social accountability within their respective medical schools. Additionally, we examined the potential relationships between gender, study year, and perceived social accountability.

The findings of our study revealed that Syrian medical students generally perceive their institutions as socially responsible and committed to the principles of social accountability, mirroring similar findings from a study conducted in the Kingdom of Saudi Arabia (KSA) [13]. However, a notable divergence emerged in the perceptions of male and female students regarding social accountability. Only 2.4% of male respondents rated themselves as having a good level of social responsibility, while a significantly higher percentage, 25%, of female participants considered themselves socially responsible individuals. This gender-based difference might be attributed to an increasing interest among female students in community involvement compared to their male counterparts.

Furthermore, our study highlighted variations in perceived social accountability among medical students in different academic years. Specifically, 10.3% of fifth-year students and 4.9% of fourth-year students stated that their school has a clear and strong social mission statement regarding the areas it serves. Additionally, 24.1% of students reported being required to participate in social services, including community-based learning.

These findings are consistent with a recent study conducted in Morocco, which indicated that students actively engaged in social service projects tend to hold a more positive view of social accountability [14]. Encouraging participation in community-based welfare programs, which are essential stakeholders in the healthcare system, and enhancing the clinical skills of medical students are believed to improve their perception of their school’s impact on delivering essential healthcare to the community [15].

However, a significant proportion of our study participants (44.3%) expressed that their educational institutions did not actively encourage them to pursue generalist specialties. This lack of support could potentially hinder the development of a more favorable perception of student involvement in social accountability initiatives. This finding resonates with a poll conducted by KSAU-US, revealing that a considerable portion of medical students lacked interest in pursuing a career in general medicine [16]. In contrast, medical students in the United Kingdom (UK) were more likely to consider general practice as a future specialty, possibly influenced by factors such as higher salaries and greater integration of general practitioners (GPs) into healthcare delivery through the National Health Service (NHS) [17]. Future research should delve into the intricate web of influences, exploring not only institutional practices but also external social factors like the standard of living and access to luxuries, to gain a more nuanced understanding of their impact on students’ perceptions of social accountability. This cautious approach ensures that assertions regarding causation are made with due consideration to the broader context and potential influencing variables.

Given the concentration of primary care facilities in rural areas, many aspiring medical professionals may encounter challenges in navigating their career paths [17]. One factor that might boost social accountability among students is their motivation and dedication to service [18]. Notably, our study found that 31% of students reported their school’s participation in community-based research.

This observation suggests a commendable trend wherein students are actively taking the initiative to conduct research in various critical areas outlined by their universities. While our study indicates a numerical representation of this participation, it’s important to clarify that this finding is a direct result derived from our data. Students’ proactive engagement in community-based research is a significant aspect that aligns with the broader emphasis on medical research, as observed in Saudi Arabia [13]. In certain institutions there, a mandatory medical research course is integrated into the curriculum during the third and fourth years of study [13].

However, it’s essential to delve deeper into the significance and potential impact of this involvement in community-based research. Beyond a numerical representation, understanding how such engagement contributes to the development of social accountability among medical students could provide valuable insights. For instance, exploring the nature of these research projects, their alignment with community needs, and the extent of student interaction with local populations could shed light on the transformative potential of student-led research initiatives. This deeper exploration would not only enhance the interpretation of our findings but also contribute to a richer understanding of the dynamics between student engagement in community-based research and the cultivation of social accountability.

### Clinical implications

The clinical implications of our study are significant. The varying perceptions of social accountability among medical students, influenced by factors such as gender and academic year, highlight the importance of tailored approaches in medical education. To promote social accountability, medical institutions should consider gender-specific initiatives and create opportunities for students to engage in community-based learning and research throughout their academic journey. Recognizing the impact of clinical experience on perceptions of social accountability underscores the value of early exposure to patient care and community engagement during medical training.

### Recommendations

In light of our study’s findings, we propose several recommendations to enhance the cultivation of social accountability among medical students. Firstly, medical institutions should implement gender-specific support programs, including mentorship initiatives and awareness campaigns, encouraging both male and female students to actively participate in community engagement and social accountability endeavors. Second, the integration of structured community-based learning experiences early in the medical curriculum is paramount. This step ensures that students are exposed to the social determinants of health and instills in them the significance of addressing community healthcare needs from the beginning of their medical education. Third, there should be a deliberate effort to encourage and facilitate medical student involvement in community-based research projects. Providing research opportunities related to healthcare disparities and social determinants of health can foster a deeper understanding of social accountability and instigate a culture of inquiry and responsibility. Fourth, medical institutions should actively promote generalist specialties, such as family medicine and general practice, as viable and rewarding career paths for medical students. Offering elective courses and clinical rotations in these specialties can stimulate interest and increase student participation, contributing to a more diverse and balanced medical workforce. Finally, longitudinal studies should be conducted to track changes in students’ perceptions of social accountability throughout their medical education, providing valuable insights into the long-term impact of educational interventions and guiding ongoing improvements in medical curricula and practices. In summary, these recommendations aim to address identified factors influencing social accountability perceptions, fostering a culture of responsibility and community engagement within the medical education framework.

### Limitations

Several limitations should be acknowledged in our study. The cross-sectional study design constrained our ability to establish causal relationships and analyze students’ perspectives, awareness, and comprehension of the concepts and principles of social accountability. Future studies with longitudinal designs could provide further insights and validate our findings. Additionally, the skewed gender representation in our sample warrants future surveys with balanced gender representation to yield more comprehensive insights into perceived social accountability. Furthermore, variations in data collection methods, statistical analyses (crude or adjusted), and differences in community engagement among various countries may limit the comparability of our results. Lastly, since our study primarily focused on medical students, future surveys should encompass insights from nurses, medical practitioners, and specialists to gauge their perspectives on social accountability.

In conclusion, our study highlights the significant influence of clinical experience and gender on Syrian medical students’ perceptions of social accountability. To enhance these perceptions, medical institutions should tailor support services for different stages of training and target initiatives to engage male students. By fostering a sense of social responsibility, medical education can contribute to a more community-oriented healthcare workforce, ultimately improving patient care and societal well-being.

## Data Availability

The authors have access to and have saved all the data necessary to support this paper’s conclusion. All data are accessible upon reasonable request from the corresponding author.

## Declarations

### Data collection group

Mohammed Taher Abras: Faculty of Medicine, Aleppo University, Aleppo, Syria. (taherabras3@gmail.com)

Douaa Oudeh: Faculty of Medicine, Damascus University, Damascus, Syria. (oudehdouaa@gmail.com)

Asaad Kandalaft: Faculty of Medicine, Damascus University, Damascus, Syria. (asaad.k20@gmail.com)

Doaa Salamah: Faculty of Medicine, Tishreen University, Lattakia, Syria. (do3a2sal2000@gmail.com)

Maryan Sakally: Faculty of Medicine, Tishreen University, Lattakia, Syria. (maryansakally5@gmail.com)

Michael Akhras: Faculty of Medicine, Damascus University, Damascus, Syria. (Micheal.akhras@gmail.com)

Kamar Alshoumary: Faculty of Medicine, Damascus University, Damascus, Syria. (kamaralshoumary@gmail.com)

Lara Aljbaee: Faculty of Medicine, Tishreen University, Lattakia, Syria. (Larajbaee@gmail.com)

Shahd Abo Ammar: Faculty of Medicine, Tishreen University, Lattakia, Syria. (shahed.rammah@gmail.com)

Kamar Algehwani: Faculty of Medicine, Al-Baath University, Homs, Syria. (qamaraljhwani123@gmail.com)
